# Stakeholder Priorities for Functional Foods Intended for Children: An AHP Evaluation of Sensory, Health, Family, and Product Attributes

**DOI:** 10.3390/foods15203611

**Published:** 2026-10-11

**Authors:** Yen-Cheng Chen, Ching-Sung Lee, Pei-Ling Tsui, Ming-Chen Chiang, Bo-Kai Lan, Mei-Yi Tsai

**Affiliations:** 1 Department of Family Science, Chinese Culture University, Taipei 111396, Taiwan; cyc4@ulive.pccu.edu.tw; 2 Department of Restaurant, Hotel and Institutional Management, Fu Jen Catholic University, New Taipei City 242062, Taiwan; 3 Department of Hospitality Management, National Taitung Junior College, Taitung 95045, Taiwan; 4 Graduate Institute of Technological and Vocational Education, National Taipei University of Technology, Taipei 10608, Taiwan; 5 Department of Food and Beverage Management, Taipei City University of Science and Technology, Taipei 112302, Taiwan; mc.chiang@tpcu.edu.tw; 6 Ph.D. Program in Nutrition and Food Science, Fu Jen Catholic University, New Taipei City 242062, Taiwan; lanbaikai@gmail.com (B.-K.L.); hlyy13954632@gmail.com (M.-Y.T.)

**Keywords:** children, functional foods, family perspective, food acceptance, sensory appeal, nutritional value, product attributes, multi-stakeholder evaluation

## Abstract

Evaluating functional foods intended for children involves nutritional and health-related value, safety, sensory qualities, and suitability for family use. This study examined the relative priorities assigned to these attributes by adult stakeholders. An online Analytic Hierarchy Process (AHP) questionnaire was administered in Taiwan to parents and four professional stakeholder groups using a framework comprising six dimensions and 18 criteria. Of 39 completed questionnaires, 35 met the predefined consistency requirement and were retained. Mean weights were calculated using aggregation of individual priorities (AIP), with standard deviations, bootstrap confidence intervals, and leave-one-out analyses used to assess variability and sensitivity. Safety and ingredient trust received the highest mean weight (0.247), followed by nutritional and functional value (0.217) and sensory appeal (0.179), together accounting for 64.3% of the total weight. Food safety ranked first among the criteria (0.113), followed by taste and flavor (0.085), functional health benefits (0.085), and ingredient quality and naturalness (0.084), with rankings based on unrounded weights. The five stakeholder profiles showed relatively high rank concordance (W = 0.739, χ^2^(5) = 18.486, *p* = 0.002). Sensory appeal ranked first among industry and product development practitioners, whereas parental and family considerations ranked second among parents. These descriptive findings provide a preliminary framework for prioritizing attributes during early-stage product evaluation. They represent adult judgments rather than children’s directly measured food acceptance and warrant product-specific assessment alongside children’s sensory responses and family purchasing and consumption behavior.

## 1. Introduction

Functional foods are positioned between conventional nutrition and health-oriented consumption because they are expected to provide benefits beyond basic nutritional requirements. Their acceptance, however, depends on more than the presence of functional ingredients or health claims. Previous research has shown that functional food acceptance reflects a combination of perceived health benefits, attitudes toward health-enhancing products, food-choice motives, and product-related characteristics [1,2,3]. This issue becomes more complex when products are developed specifically for children. Children are the direct consumers of these foods, yet parents commonly influence what is purchased, provided, and incorporated into household diets. Annunziata et al. [4] demonstrated the relevance of parental evaluations in choices involving functional foods targeted at children, while more recent studies have continued to situate children’s functional food consumption within the combined context of children’s eating habits and parental knowledge and perceptions [5,6]. Accordingly, the evaluation of functional foods intended for children involves both characteristics relevant to children’s potential acceptance and judgments made by adults who purchase, provide, develop, or evaluate these products. These adult judgments should be distinguished from children’s directly observed sensory or behavioral acceptance.

For children, sensory experience represents one of the most immediate bases of food acceptance. Healthy or nutritionally enhanced foods are unlikely to be repeatedly consumed if their sensory characteristics do not correspond with children’s preferences, and these preferences are shaped by several dimensions rather than taste alone [7]. Texture has received particular attention because oral processing, softness, crispness, smoothness, and other mouthfeel characteristics can differ substantially in their acceptability across children and developmental stages [8,9,10,11]. Visual characteristics and overall flavor also contribute to how children approach and evaluate unfamiliar or reformulated foods, while food neophobia and sensitivity to novel sensory cues may further limit willingness to try new products [12,13]. Recent work comparing children and adults has likewise shown that sensory and emotional responses remain relevant to the acceptance of emerging food products [14]. These findings provide a basis for including taste and flavor, texture and mouthfeel, and appearance and color as distinct sensory criteria. In the present study, however, their importance is evaluated through adult stakeholder judgments rather than direct sensory testing with children.

Sensory appeal alone does not define the perceived value of a functional food intended for children. Such products may also be evaluated according to their nutritional contribution, additional health-related properties, and suitability for children at different developmental stages [3,15]. Parents may consider not only nutritional value but also whether the claimed benefits are relevant and appropriate for children [4,5,6]. Nutritional value, functional health benefits, and age-appropriate nutritional suitability were therefore treated as related but distinguishable criteria. The first concerns the product’s basic dietary contribution, the second concerns benefits beyond basic nutrition, and the third concerns whether the nutritional profile is considered appropriate for the intended age group.

Parents also shape the household setting in which children’s food preferences are translated into purchasing and provision decisions. Children’s preferences interact with parental priorities, feeding practices, food availability, health concerns, and everyday meal routines [16,17,18,19,20]. Decisions concerning unfamiliar, reformulated, or alternative foods may therefore reflect both children’s responses and caregivers’ evaluations [21,22,23]. Within the present framework, parental health expectations, compatibility with family eating practices, and parental willingness to provide the product represent different aspects of this family decision context. Parental willingness was retained as a separate criterion because it refers to the adult’s eventual provision judgment, whereas the remaining criteria refer to particular product attributes or household conditions that may inform that judgment.

The evaluation of foods intended for children also begins before consumption. Parents and other adult decision makers rely on information concerning safety, ingredients, packaging, health claims, and product quality when judging whether a product is suitable for children. Parent-directed claims and front-of-pack imagery may influence perceptions of healthfulness, suitability, and purchase intention [24,25,26], while packaging design and quality cues affect how products are interpreted in the absence of direct consumption experience [27,28,29]. Portion size, product form, accessibility, convenience, and affordability may further affect whether a product can be incorporated into everyday family practices [30,31]. These considerations support distinguishing safety and ingredient trust, packaging and product communication, and practical and economic value from sensory and nutritional evaluation.

The existing literature provides evidence concerning sensory characteristics, nutritional and functional value, family food decisions, safety, packaging, and practical usability, but these considerations are generally examined within separate research traditions. Consequently, it remains unclear how adult stakeholders prioritize these attributes when they are evaluated simultaneously and whether similar priority patterns emerge across stakeholder groups. The Analytic Hierarchy Process (AHP) is suited to this type of problem because it allows multiple attributes to be compared in relative rather than isolated terms. AHP has been applied in food research to preference elicitation, sensory characterization, organic food choice, innovative food product development, and consumer-oriented multicriteria evaluation [32,33,34,35,36]. It therefore provides an appropriate structure for estimating stakeholder-assigned priorities without interpreting those priorities as observed determinants of children’s actual acceptance.

This study examines how adult stakeholders prioritize attributes relevant to the evaluation of functional foods intended for children. The evaluation framework includes six dimensions: sensory appeal, nutritional and functional value, safety and ingredient trust, packaging and product communication, parental and family considerations, and practical and economic value. These dimensions are represented by 18 criteria and organized within a hierarchical decision structure. The dimensions and criteria were derived from the literature and subsequently reviewed through expert discussion to confirm their relevance, conceptual distinction, and suitability for pairwise comparison. Using AHP, the study estimates overall priorities for the six dimensions and 18 criteria and compares dimension-level priority patterns across the five stakeholder groups. Two research questions guide the analysis:

RQ1. What relative priorities do adult stakeholders assign to the six dimensions and 18 criteria used to evaluate functional foods intended for children?

RQ2. How similar or different are the dimension-level priority patterns assigned by parents and the four professional stakeholder groups?

The analysis therefore concerns stakeholder-perceived priorities within a common evaluation framework rather than children’s directly measured food acceptance.

## 2. Literature Review

### 2.1. Children’s Food Acceptance and Sensory Evaluation

Food acceptance in childhood is strongly shaped by sensory experience, including flavor, texture, appearance, familiarity, and prior eating experience, with responses varying across individuals and developmental stages [7,37]. Children may be less willing to consume unfamiliar foods when their sensory properties differ from established preferences [38,39], a concern that is particularly relevant to functional foods because reformulation, fortification, alternative ingredients, or novel product formats may alter the eating experience despite favorable nutritional or health value.

Texture represents an important source of variation in children’s food responses. Individual differences in texture preference and sensitivity have been documented across children [8,9], while early childhood studies show that oral texture perception reflects interactions among food structure, oral processing, and sensory sensitivity [10,11]. Taste, appearance, and familiarity also influence willingness to approach and consume unfamiliar foods. Food neophobia may amplify avoidance of novel sensory cues [12], and evidence from insect-based, plant-based, and other emerging foods indicates that sensory and emotional responses interact with product novelty in shaping acceptance [13,14,40,41]. These findings support treating taste and flavor, texture and mouthfeel, and appearance and color as related but distinct components of sensory appeal.

Sensory evaluation therefore needs to consider how multiple product cues operate together rather than treating individual attributes in isolation. Drake et al. [42] emphasized the importance of integrating sensory measurement with consumer preference, while research in other food contexts has shown that evaluation can extend beyond taste to visual, tactile, and packaging-related cues [43]. For functional foods intended for children, comparative prioritization of sensory attributes is particularly useful because it allows their relative importance to be evaluated within a broader product framework rather than assumed from any single sensory characteristic. Nevertheless, the present study does not directly measure children’s sensory responses. Instead, this literature provides the conceptual basis for examining the importance that adult stakeholders assign to sensory attributes when evaluating foods intended for children.

### 2.2. Nutritional, Functional, and Trust-Related Evaluations

Functional food acceptance depends not only on the presence of health-enhancing properties but also on how consumers perceive their relevance, credibility, and fit with existing food-choice motives [1,2,3,44]. Functional foods may provide physiological or health-related benefits beyond basic nutrition, yet these benefits are meaningful only when consumers regard them as appropriate to the intended use context [15].

For products intended for children, evaluation extends beyond nutrient content to whether nutritional and functional benefits are appropriate for children and suitable for regular consumption. Parental choices are shaped by multiple product-related and health-related considerations rather than functional claims alone [4], while parental knowledge, attitudes, and dietary practices also influence how such foods are understood and used [5,6]. Research on food literacy further indicates that nutritional and health information should be considered in relation to children’s developmental stage [45]. These findings support distinguishing three nutritional and health-related criteria. Nutritional value refers to the product’s contribution to the child’s general dietary needs. Functional health benefits refer to additional physiological or health-related properties beyond basic nutrition. Age-appropriate nutritional suitability refers to whether the nutritional composition and intended benefits are considered appropriate for the child’s developmental stage.

Trust-related judgments form a separate dimension. Consumers assess ingredient quality, production characteristics, safety, and external quality signals alongside nutritional and functional value [29,46]. In products intended for children, ingredient naturalness, additives, raw-material quality, certification, and quality assurance may contribute to confidence in product suitability and credibility. Food safety concerns potential risks associated with consumption, ingredient quality and naturalness concern the perceived characteristics and composition of the ingredients, and certification and quality assurance concern external evidence that supports product reliability.

Separating nutritional and functional value from safety and ingredient trust prevents all health-related attributes from being treated as a single construct. The distinction also allows adult stakeholders to indicate whether they give greater priority to what a product is expected to provide nutritionally or to whether the product is regarded as safe and dependable. These priorities remain stakeholder evaluations and should not be interpreted as evidence of children’s actual dietary or behavioral responses.

### 2.3. Family Decision Context, Product Communication, and Everyday Usability

Children’s food acceptance is embedded in household decision making rather than determined by the child alone. Preferences, requests, and refusals interact with parental control, food availability, feeding practices, and everyday family circumstances [16,17,47]. Parental food-choice motives, perceptions of the food environment, socioeconomic constraints, home food availability, and role modeling further shape which foods are purchased and incorporated into children’s diets [18,19,20,48,49]. Food provision is therefore negotiated within a family context in which children’s preferences and parental judgments operate together.

Recent studies reinforce this interactive characteristic of family food decisions. Children and caregivers may differ in their preferences for alternative or unfamiliar foods [21], while parental choices for young children can involve convenience, health perceptions, and everyday practical conditions [22]. Parents may also evaluate emerging foods differently when deciding for themselves and for their children [23], and children can actively participate in household consumption decisions [50]. These findings position family food choice as a process of balancing child acceptance, parental priorities, and practical household demands.

Within this family decision context, parental health expectations, compatibility with family eating practices, and parental willingness to provide the product represent related but distinct judgments. Parental health expectations concern the benefits that parents believe a product should provide. Compatibility with family eating practices concerns whether the product fits established household routines and dietary patterns. Parental willingness to provide refers to the parent’s stated readiness to make the product available to the child. Although this willingness may be informed by safety, nutrition, price, convenience, and sensory expectations, it was retained as a separate criterion because it represents the provision judgment through which these considerations may be translated into household use. It is not treated as a direct measure of either purchasing behavior or children’s acceptance.

Product communication contributes to this process before consumption. Packaging, health claims, visual imagery, and nutrition information can shape parental expectations and purchase intentions for foods intended for children [24,25,26]. Packaging aesthetics and informational cues also influence product evaluation more broadly [27,28], while evidence from other food contexts indicates that visual and packaging-related cues may extend evaluation beyond taste alone [43]. Packaging and visual design concern the product’s external presentation, clarity of product information concerns whether the information can be readily understood, and credibility of health claims concerns whether the communicated benefits are regarded as trustworthy.

Practical suitability further affects whether a product can be adopted within everyday family life. Portion size, age appropriateness, accessibility, convenience, and ease of use all enter parental evaluations of foods for children [30,51,52,53]. Child-centered product development research also supports incorporating children’s experiences into product design [31]. Accordingly, child-appropriate product format, convenience of purchase and use, and affordability and value for money were included as separate components of practical and economic value. Together, family considerations, product communication, and practical value capture the conditions under which a product judged favorably in principle might be considered suitable for routine household provision.

### 2.4. A Multi-Stakeholder Prioritization Framework for Functional Foods Intended for Children

Children’s food acceptance involves sensory experience, nutritional and health value, safety and trust, family decision making, product communication, and practical use. These considerations have often been examined separately across sensory, family, functional food, and packaging research, although food choice itself reflects multiple interacting influences [46]. Examining these domains in isolation makes it difficult to determine their relative importance when they must be considered simultaneously.

AHP provides a structured approach for comparing such competing criteria through pairwise judgments. Its application in food research has included preference elicitation, sensory characterization, organic food choice, innovative food product development, and consumer-oriented multicriteria evaluation [32,33,34,35,36]. These studies demonstrate the usefulness of relative weighting when several product attributes must be evaluated within the same decision framework. In the present study, AHP is used to estimate the priorities assigned by adult stakeholders rather than to identify causal determinants of children’s food acceptance.

The context of functional foods intended for children also requires consideration of whether stakeholder groups assign similar priorities to the six evaluation dimensions. Parents contribute household purchasing and food-provision experience, whereas academic researchers, food and nutrition professionals, food industry or product development practitioners, and child health, education, family, or related practitioners contribute perspectives derived from their respective fields. The comparison of these groups is intended to describe similarities and differences in their priority patterns within the present sample. It is not intended to establish statistically generalizable differences between broader stakeholder populations.

Drawing on the preceding literature, the evaluation framework was organized into six dimensions: sensory appeal, nutritional and functional value, safety and ingredient trust, packaging and product communication, parental and family considerations, and practical and economic value. Each dimension contains three conceptually defined criteria, resulting in 18 criteria for pairwise comparison. The preliminary framework was subsequently reviewed through expert discussion to examine the relevance, clarity, and conceptual distinction of the dimensions and criteria. This process was used to refine the wording and organization of the framework, although it was not treated as a formal psychometric content-validity test.

The resulting hierarchy enables adult stakeholders to compare sensory, health, family, communication, and practical considerations within a common decision structure. It therefore provides a framework for examining stakeholder-perceived priorities concerning functional foods intended for children while maintaining a clear distinction between adult evaluations and children’s directly observed acceptance.

## 3. Materials and Methods

### 3.1. Research Design and Analytical Framework

This study employed the Analytic Hierarchy Process (AHP) to estimate the relative priorities that adult stakeholders assigned to attributes used in evaluating functional foods intended for children. AHP is a multi-criteria decision-making approach that structures complex evaluation problems hierarchically and derives relative priorities through pairwise comparisons [54]. It was selected because the research problem involves multiple attributes that cannot be meaningfully evaluated through isolated importance ratings alone. The method enables respondents to make pairwise judgments between competing criteria and converts these judgments into relative priority weights within a hierarchical structure. This approach is particularly appropriate for the present study because sensory, nutritional, safety, family, communication, and practical considerations may all contribute to product evaluation, while their relative importance remains the primary analytical interest.

The study was exploratory and judgment-based. It was designed to identify how adult stakeholders prioritized sensory, nutritional, safety, family, communication, practical, and economic considerations. It did not directly assess children’s sensory responses, actual food intake, purchasing behavior, or acceptance of a specific product. Accordingly, the resulting weights were interpreted as stakeholder priorities within the predefined evaluation framework rather than as estimates of children’s actual behavioral responses.

The evaluation framework was organized into three hierarchical levels. The first level represented the overall decision goal: prioritizing adult stakeholder evaluations of attributes associated with functional foods intended for children. The second level consisted of six evaluation dimensions: sensory appeal, nutritional and functional value, safety and ingredient trust, packaging and product communication, parental and family considerations, and practical and economic value. Each dimension was further divided into three criteria, resulting in 18 criteria at the third level. The hierarchical structure allowed priorities to be estimated both within each dimension and across the complete evaluation framework.

**Figure 1:**
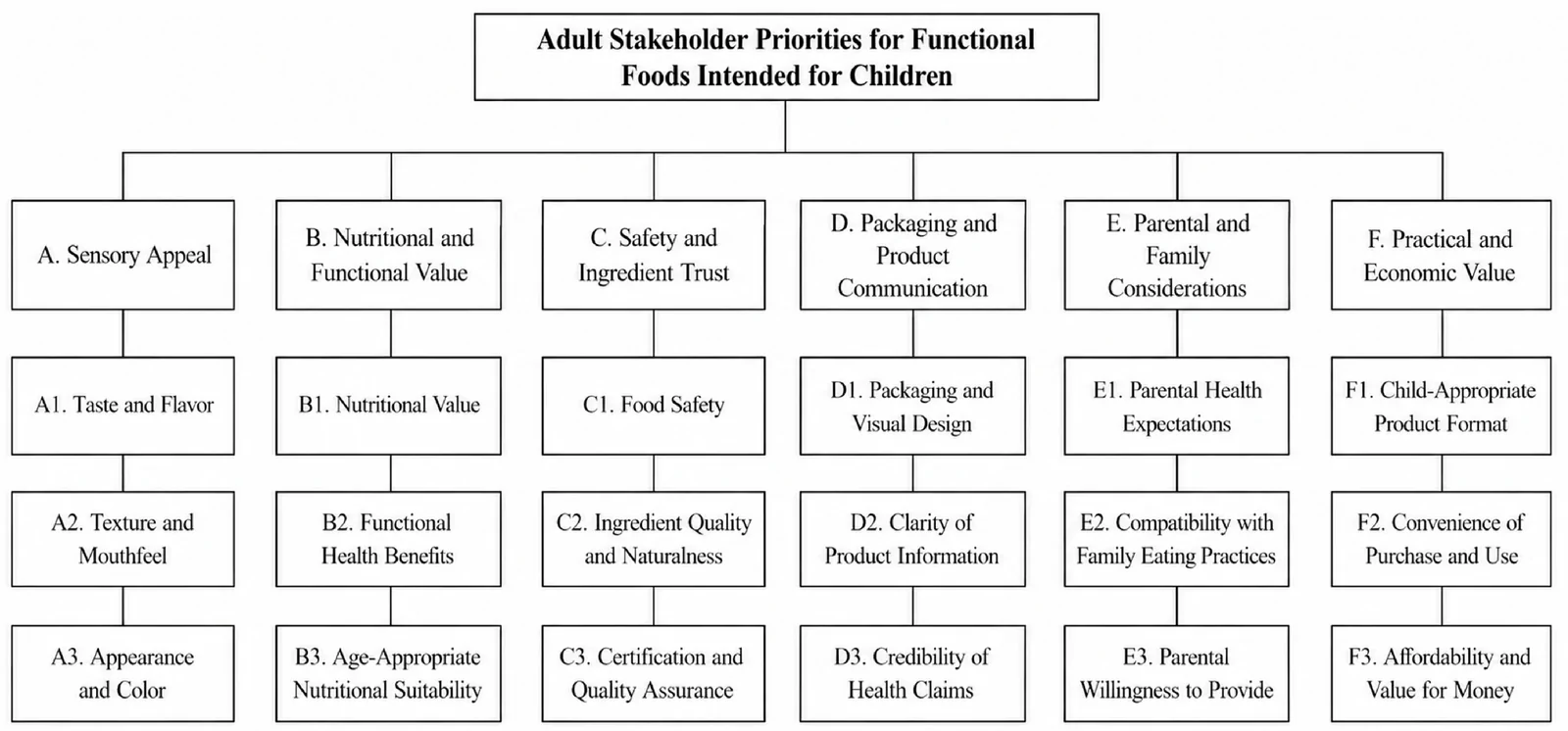
Hierarchical framework for prioritizing adult stakeholder evaluations of functional foods intended for children. Note: The framework consists of one overall goal, six evaluation dimensions, and 18 criteria used for AHP pairwise comparisons and priority estimation. The resulting priorities represent adult stakeholder judgments and do not constitute direct assessments of children’s food acceptance or consumption behavior.

The study adopted a multi-stakeholder design rather than relying on a single respondent group. Judgments were obtained from five adult stakeholder groups: parents, academic researchers, food and nutrition professionals, industry and product-development practitioners, and child health, education, and family-related practitioners. Parents contributed experience concerning household food purchasing, provision, and children’s everyday eating practices. The four professional groups contributed perspectives informed by research, nutritional practice, product formulation and development, child health, education, and family-related services. This design was intended to capture the relative importance assigned to the same set of attributes from family and professional perspectives without assuming that stakeholder groups would necessarily prioritize them in the same way. The same hierarchical structure and pairwise comparison procedure were used for all five groups, allowing their priority patterns to be examined within a common evaluation framework. The degree of convergence or divergence among the groups was treated as an empirical question rather than assumed in advance. Figure 1 presents the hierarchical evaluation framework used in the study.

### 3.2. Development of the Evaluation Criteria

The evaluation criteria were derived from the literature reviewed in the preceding sections and organized to represent conceptually distinct aspects of child-oriented functional food evaluation. The framework was not intended to reproduce every determinant previously associated with children’s eating behavior. Instead, the criteria were limited to attributes that could be meaningfully evaluated as characteristics of the product or its family consumption context and compared through pairwise judgments.

After the preliminary hierarchy had been developed, the six dimensions, 18 criteria, operational definitions, and questionnaire wording were examined through expert discussion and review. The expert panel comprised 13 specialists whose expertise covered food science and nutrition, consumer behavior and marketing, family science and family education, child development and education, and food product development and hospitality management. The review considered the relevance and coverage of the proposed criteria, the clarity of their wording, and the conceptual distinction among related elements. The framework was refined in response to this process, and the six dimensions and 18 criteria were confirmed before questionnaire administration. This procedure constituted an expert review of the proposed evaluation structure and questionnaire rather than a direct validation of children’s food responses.

The sensory appeal dimension captured the sensory characteristics considered relevant to children’s anticipated experience of the product through taste and flavor, texture and mouthfeel, and appearance and color. Nutritional and functional value distinguished conventional nutritional contribution from additional health-related benefits and the suitability of the nutritional profile for children. Safety and ingredient trust represented judgments concerning food safety, ingredient quality and naturalness, and certification or quality assurance. These criteria were treated separately from nutritional value because perceived safety and trust concern confidence in the suitability and reliability of the product rather than the nutritional benefits it provides. Within this dimension, food safety referred to potential risks associated with ingredients, production, storage, and consumption. Ingredient quality and naturalness concerned the perceived characteristics of the ingredients, whereas certification and quality assurance represented external signals that could support confidence in the product.

Packaging and product communication reflected the external information through which the product is evaluated before consumption, including packaging and visual design, clarity of product information, and credibility of health claims. This dimension concerned how product information and visual cues were presented and interpreted, rather than whether the ingredients or production process were themselves considered safe. Parental and family considerations captured the household context in which child-oriented foods are selected and provided. The three criteria addressed parental health expectations, compatibility with established family eating practices, and parental willingness to provide the product. Practical and economic value represented conditions affecting routine use, including child-appropriate product format, convenience of purchase and use, and affordability and value for money.

Parental willingness to provide was retained as a family provision consideration because parents commonly act as gatekeepers in the purchase and provision of foods intended for children. This criterion was more decision-proximal than parental health expectations or compatibility with family eating practices. However, it was not treated as evidence of children’s acceptance, actual parental purchasing behavior, or routine product consumption.

Conceptual overlap among the criteria was minimized by defining each criterion according to a specific evaluative function. For example, nutritional value referred to the nutritional contribution of the product, whereas functional health benefits referred to benefits beyond basic nutrition. Age-appropriate nutritional suitability addressed whether the nutritional profile and intended use corresponded with children’s age and developmental stage. Food safety concerned potential risks associated with consumption, while ingredient quality and naturalness reflected characteristics of the ingredients themselves. Packaging and product communication addressed how the product and its information were presented, whereas safety and ingredient trust concerned confidence in the product’s safety and integrity. Parental and family considerations addressed parental expectations and compatibility with household food practices, whereas practical and economic value addressed operational feasibility and household resource requirements.

Parental willingness to provide was distinguished from children’s acceptance because it represented the parent’s provision decision rather than a direct measure of the child’s response. This distinction was particularly important because children were not surveyed directly in the present study. Accordingly, criteria referring to children’s likely preferences or suitability were evaluated through adult stakeholder judgments and should not be interpreted as direct reports from children. The six dimensions, 18 criteria, and their corresponding operational definitions are presented in Table 1.

### 3.3. Participants and Expert Recruitment

A purposive stakeholder sampling strategy was adopted because the study sought to elicit informed judgments and prioritize evaluation criteria rather than estimate population prevalence or examine causal relationships. Participants were recruited from stakeholder groups with relevant academic, professional, practical, or household experience related to children’s food consumption and functional foods. The professional participants included academic researchers, food and nutrition professionals, food industry and product development practitioners, and professionals working in child health, education, family-related services, or other closely related fields. Eligible professional participants were required to have at least 15 years of relevant academic, professional, or practical experience.

The 15-year threshold was established a priori to operationalize sustained domain familiarity. This criterion was informed by the AHP emphasis on selecting judges who are experienced and knowledgeable in the relevant domain and by previous AHP research that used at least 15 years of relevant experience as an eligibility requirement for expert respondents [55,56]. It was applied as a study-specific eligibility criterion for professional participants rather than as a universal requirement for AHP research.

Parents were included as a separate stakeholder group to incorporate judgments grounded in everyday household food purchasing and provision. Eligible parents were required to have at least one child under 12 years of age and to be directly involved in decisions concerning children’s food purchase, selection, or provision. Their inclusion was intended to provide a family-based perspective that complemented the professional and academic judgments represented in the study. Parents were not required to meet the 15-year professional experience criterion because their eligibility was based on current caregiving responsibilities and direct involvement in household food provision.

Recruitment and data collection were conducted in Taiwan in July 2026 using an online questionnaire. Participants were recruited primarily through purposive sampling, supplemented where appropriate by referrals to other eligible participants with relevant expertise or experience. The sampling process sought to maintain diversity across disciplinary and practical backgrounds rather than concentrate respondents within a single professional field. Respondents identified the stakeholder category that best represented their primary role. Professional participants also reported their field of expertise and years of relevant experience, while parents provided information concerning their child’s age and their involvement in household food decisions.

A total of 39 eligible participants were invited to participate in the study, and all 39 began and completed the online questionnaire, yielding a response rate of 100%. No incomplete or partially completed questionnaires were recorded. Four completed questionnaires were excluded from the AHP analysis because their pairwise comparison judgments did not satisfy the predefined consistency requirement of CR ≤ 0.10, as detailed in Section 3.5. All 35 questionnaires retained for analysis met the consistency requirement, representing an analytical retention rate of 89.7% among the 39 completed questionnaires. The final sample comprised 8 parents, 6 academic researchers, 6 food and nutrition professionals, 7 food industry or product development practitioners, and 8 professionals from child health, education, family-related, or other relevant fields.

Although the final sample was modest, its adequacy should be evaluated in relation to the judgment-based design of AHP rather than conventional statistical power criteria. AHP derives priority weights from structured comparisons made by knowledgeable participants and therefore does not require a large probability sample. Darko et al. [57] noted that AHP has no fixed minimum sample-size requirement and identified its suitability for small, expertise-based panels as a methodological advantage. Earlier studies likewise used relatively small expert panels to develop consistent decision models [58,59]. Accordingly, the 35 qualified participants distributed across five stakeholder groups, all of whom met the CR ≤ 0.10 requirement, provided an appropriate basis for the present exploratory prioritization. Nevertheless, the findings represent sample-specific stakeholder judgments and should not be generalized as population-level estimates [57,60]. The composition of the final analytical sample is presented in Table 2.

### 3.4. AHP Questionnaire and Pairwise Comparison Procedure

The AHP questionnaire was developed on the basis of the hierarchical evaluation framework established in the preceding literature review. Before formal administration, the draft questionnaire was reviewed by experts to examine the relevance and clarity of the dimension and criterion descriptions, the comprehensibility of the instructions, and the usability of the pairwise comparison format. The six dimensions and 18 criteria were confirmed following this review.

Before completing the pairwise comparisons, participants were provided with a definition of child-oriented functional foods together with brief descriptions of the six dimensions and 18 evaluation criteria. Child-oriented functional foods were defined as conventional food products intended primarily for children that provide basic nutrition while offering additional health or physiological benefits. Medicines and products primarily consumed in supplement forms, such as capsules or tablets, were excluded. Participants were instructed to evaluate functional foods as a general category of health-oriented foods that children may consume in everyday life rather than with reference to a specific brand or product. They were also informed that the task concerned their own assessment of the relative importance of the attributes and did not require them to report children’s actual sensory responses or consumption behavior.

The questionnaire contained two levels of pairwise comparison. At the dimension level, the six major dimensions were compared with one another, generating 15 pairwise judgments. At the criterion level, the three criteria within each dimension were compared pairwise. Each three-criterion set generated three comparisons, resulting in 18 criterion-level judgments across the six dimensions. The questionnaire therefore comprised 33 pairwise comparisons in total. This structure enabled the relative importance of both the major dimensions and their corresponding criteria to be estimated within the same hierarchical framework.

The online questionnaire included written completion instructions and a worked example illustrating how to indicate equal importance and different levels of preference between two factors. All participants received the same instructions and examples. The dimensions and criteria were presented in a fixed order, and the same sequence of pairwise comparisons was used for every participant. No randomization or counterbalancing of the comparison order was applied.

Each pairwise judgment was made using Saaty’s nine-point comparison scale [54]. A value of 1 indicated equal importance between the two factors, whereas values of 3, 5, 7, and 9 represented increasing levels of relative importance, ranging from moderate to extreme preference. Intermediate values of 2, 4, 6, and 8 were available when a participant’s judgment fell between two adjacent levels. To reduce ambiguity in responding, the names of the two factors being compared were displayed on opposite sides of the scale, with the value 1 positioned at the midpoint. Participants were instructed to select one value for each comparison based on their professional, practical, or household experience.

Responses were subsequently converted into reciprocal pairwise comparison values in accordance with the AHP procedure [54]. When the factor displayed on the left side of the scale was judged more important, the selected value was entered directly into the comparison matrix. When the factor displayed on the right side was judged more important, the reciprocal of the selected value was used. For example, a preference intensity of 5 in favor of the left-side factor was coded as 5, whereas the same preference intensity in favor of the right-side factor was coded as 1/5. A judgment of equal importance was coded as 1. The same coding procedure was applied consistently to all dimension-level and criterion-level comparisons.

### 3.5. AHP Weight Estimation and Consistency Assessment

Individual AHP calculations were performed using Expert Choice software (version 11.5). Pairwise comparison judgments were organized into reciprocal matrices for each participant. For a comparison matrix *A* = (*a_ij_*), the element *a_ij_* represented the relative importance assigned to element *i* compared with element *j*, with *a_ji_* = 1/*a_ij_*. The diagonal elements were set to 1 [54].

Priority weights were derived using the principal eigenvector method [54]. The normalized priority vector **w** satisfied

$$Aw=\lambda_{\max}w,\;\sum_{i=1}^{n}w_{i}=1,$$

where *λ*_max_ represents the maximum eigenvalue of the comparison matrix. Dimension weights expressed relative importance with respect to the overall evaluation goal, whereas local criterion weights expressed relative importance within each dimension. For each participant, the global weight of a criterion was calculated by multiplying its local weight by that participant’s weight for the corresponding dimension.

Consistency was assessed before the individual priorities were summarized. The Consistency Index (CI) and Consistency Ratio (CR) were calculated as follows [54]:

$$\mathrm{CI}=\frac{\lambda_{\max}-n}{n-1},$$

$$\mathrm{CR}=\frac{\mathrm{CI}}{\mathrm{RI}},$$

where *n* represents the number of elements in the comparison matrix and *RI* is the corresponding Random Index. The RI values were 0.58 for the three-element criterion matrices and 1.24 for the six-element dimension matrix. A CR of 0.10 or lower was considered acceptable [54].

The dimension-level matrix and each of the six criterion-level matrices were assessed separately for every participant. A questionnaire was retained only when all seven matrices satisfied *CR* ≤ 0.10. Of the 39 completed questionnaires, four contained one or more matrices exceeding this threshold and were excluded in their entirety. The remaining 35 questionnaires yielded 35 dimension-level matrices and 210 criterion-level matrices, comprising 245 retained comparison matrices.

Two aggregation procedures served distinct purposes [61]. For aggregated consistency diagnostics, valid pairwise judgments were combined within each stakeholder group and across the overall sample using the geometric mean. This aggregation of individual judgments preserved the reciprocal structure of the matrices and allowed aggregated CR values to be calculated. These matrices were used only for consistency diagnostics and did not determine the mean priority weights reported in Tables 3–5.

The reported weights were calculated using aggregation of individual priorities [61]. This approach was selected because participants completed the questionnaire independently, without discussion or an attempt to reach a collective decision. Individual priority vectors were therefore retained before arithmetic averaging, rather than deriving a common priority vector from aggregated judgments.

For stakeholder group *g*, containing *n_g_* retained participants, the mean weight of dimension *d* was calculated as

$${\bar{w}}_{\mathrm{gd}}=\frac{1}{n_{g}}\sum_{r=1}^{n_{g}}w_{\mathrm{rd}},$$

where *w_rd_* represents the weight assigned to dimension *d* by participant *r*. Each participant contributed equally to the group mean. Overall weights were calculated by averaging the individual priority vectors of all retained participants. At the overall-sample level, mean weights were calculated for the six dimensions, the local criteria within each dimension, and the 18 global criterion weights. Mean global weights were obtained by averaging individual global weights, rather than multiplying mean dimension weights by mean local weights. Stakeholder-specific comparisons were restricted to the six dimensions. All rankings were determined using unrounded mean weights.

Standard deviations summarized variability in individual dimension and global criterion weights. For the overall dimension and global criterion means, percentile-based 95% bootstrap confidence intervals were estimated using 10,000 resamples. Participants were resampled with replacement, and the 2.5th and 97.5th percentiles of the resulting distributions defined the confidence limits. These intervals described uncertainty around the overall mean weights and were not used to test differences between stakeholder groups.

A leave-one-out sensitivity analysis assessed the influence of individual participants on stakeholder-specific dimension weights and rankings. Each participant was omitted from the relevant group in turn, and the mean weights and rankings of the six dimensions were recalculated. Changes were evaluated against the complete-group results using rank changes and the maximum absolute change in mean dimension weights.

Stakeholder comparisons were descriptive and examined the mean weights, standard deviations, and rankings of the six dimensions. Criterion-level priorities were reported for the overall sample rather than compared across stakeholder groups. To supplement the dimension-level comparisons, Kendall’s coefficient of concordance *W* quantified concordance among the five group-level rankings [62]:

$$W=\frac{12S}{m^{2}(k^{3}-k)},$$

where *m* represents the number of stakeholder groups, *k* the number of ranked dimensions, and *S* the sum of squared deviations of the dimension rank sums from their mean. The chi-square approximation was calculated as

*χ*^2^ = *m*(*k* − 1)*W*,

with *k* − 1 degrees of freedom. Kendall’s *W* described concordance among the group-level rankings derived from AIP mean weights. It did not measure agreement among individual participants or test differences between stakeholder populations. Formal tests of between-group differences were not conducted.

## 4. Results

### 4.1. Participant Characteristics

All 39 invited participants completed the online questionnaire, corresponding to a response rate of 100%. Four completed questionnaires were excluded because one or more individual pairwise comparison matrices exceeded the predefined consistency threshold of CR > 0.10. The final analysis therefore included 35 valid questionnaires. Following consistency screening, 35 dimension-level matrices and 35 criterion-level matrices for each of the six dimensions were retained, yielding 245 individual comparison matrices in total. All retained matrices satisfied the predefined requirement of CR ≤ 0.10 and were included in the calculation and aggregation of individual priority weights.

All professional participants met the eligibility requirement of at least 15 years of relevant academic, professional, or practical experience. Their backgrounds included food science, nutrition, consumer behavior and marketing, family science and family education, child development and education, public health and health promotion, culinary product development, and food service management. Parents were eligible if they had at least one child under 12 years of age and were directly involved in household decisions concerning children’s food purchase, selection, or provision.

The sample was designed to obtain informed judgments from relevant stakeholder groups rather than to statistically represent broader parent or professional populations. Accordingly, the following results describe the priority patterns of the participants retained in the present AHP analysis.

### 4.2. Overall Priority Weights of the Six Evaluation Dimensions

Table 3 presents the mean priority weights, variability, and rankings assigned to the six evaluation dimensions by the 35 retained participants using the AIP approach. Safety and ingredient trust received the highest mean weight (0.247), followed by nutritional and functional value (0.217) and sensory appeal (0.179). Together, these three dimensions accounted for 64.3% of the total priority weight. Parental and family considerations ranked fourth (0.153), followed by practical and economic value (0.110) and packaging and product communication (0.095).

**Figure 2:**
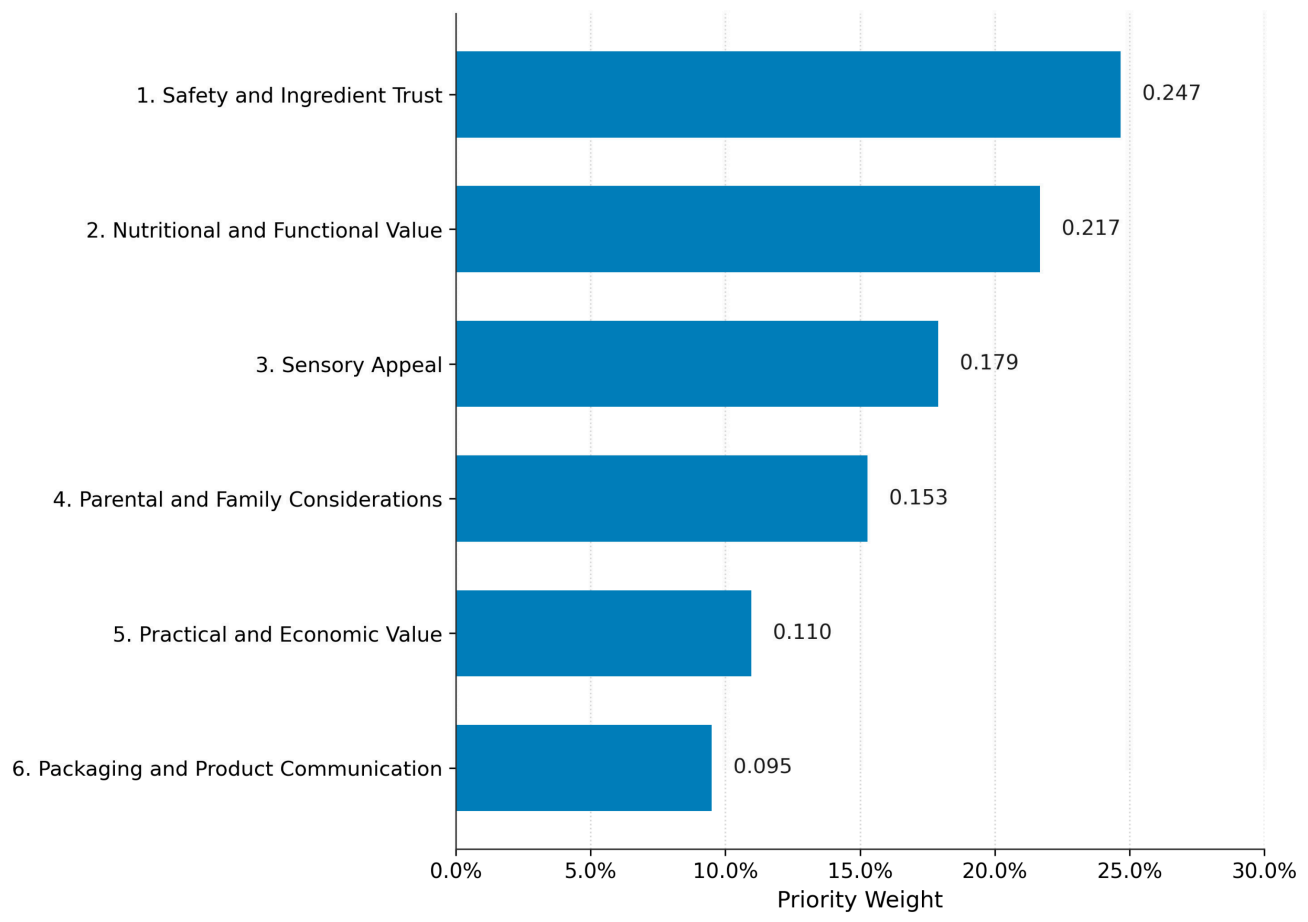
Mean priority weights assigned by adult stakeholders to the six dimensions used to evaluate functional foods intended for children.

The standard deviations and bootstrap confidence intervals indicate that individual priorities varied within the sample, particularly for parental and family considerations and sensory appeal. Nevertheless, the mean-weight ordering placed safety and ingredient trust, nutritional and functional value, and sensory appeal in the first three positions. These weights represent relative priorities within the specified AHP hierarchy and should not be interpreted as observed effects on children’s actual food acceptance. Figure 2 presents the mean priority weights across the six dimensions; the corresponding standard deviations and 95% bootstrap confidence intervals are reported in Table 3.

**Table 3:** Overall Mean Priority Weights, Variability, and Rankings of the Six Evaluation Dimensions.

Dimension	Mean Priority Weight	SD	95% Bootstrap CI	Rank
Safety and Ingredient Trust	0.247	0.048	[0.231, 0.262]	1
Nutritional and Functional Value	0.217	0.048	[0.201, 0.232]	2
Sensory Appeal	0.179	0.055	[0.162, 0.198]	3
Parental and Family Considerations	0.153	0.065	[0.132, 0.175]	4
Practical and Economic Value	0.110	0.031	[0.100, 0.120]	5
Packaging and Product Communication	0.095	0.033	[0.085, 0.106]	6
Total	1.000	—	—	—

Note. Mean weights were derived by AIP from 35 individual priority vectors, each normalized to sum to 1.000. SD = standard deviation; CI = confidence interval. The 95% CIs were estimated using 10,000 percentile bootstrap resamples.

### 4.3. Mean Local and Global Priority Weights of the Evaluation Criteria

Table 4 presents the mean local and global priority weights of the 18 evaluation criteria based on the individual priority vectors of the 35 retained participants. At the global level, food safety ranked first (0.113), followed by taste and flavor (0.085), functional health benefits (0.085), and ingredient quality and naturalness (0.084). Nutritional value (0.076) and parental health expectations (0.064) also ranked among the six highest-priority criteria. These criteria represented the safety, sensory, nutritional, and family-related dimensions rather than being concentrated within a single dimension.

**Table 4:** Mean Local and Global Priority Weights of the 18 Evaluation Criteria.

Dimension	Criterion	Mean Local Weight	Mean Global Weight	SD	95% Bootstrap CI	Global Rank
Sensory Appeal	Taste and Flavor	0.476	0.085	0.028	[0.077, 0.095]	2
	Texture and Mouthfeel	0.313	0.056	0.020	[0.050, 0.062]	8
	Appearance and Color	0.211	0.038	0.016	[0.033, 0.044]	13
Nutritional and Functional Value	Nutritional Value	0.349	0.076	0.022	[0.068, 0.083]	5
	Functional Health Benefits	0.389	0.085	0.025	[0.077, 0.093]	3
	Age-Appropriate Nutritional Suitability	0.262	0.056	0.017	[0.051, 0.062]	7
Safety and Ingredient Trust	Food Safety	0.457	0.113	0.029	[0.104, 0.123]	1
	Ingredient Quality and Naturalness	0.343	0.084	0.024	[0.076, 0.092]	4
	Certification and Quality Assurance	0.200	0.050	0.015	[0.045, 0.054]	10
Packaging and Product Communication	Packaging and Visual Design	0.220	0.021	0.008	[0.018, 0.024]	18
	Clarity of Product Information	0.352	0.034	0.014	[0.029, 0.038]	16
	Credibility of Health Claims	0.428	0.040	0.016	[0.036, 0.046]	12
Parental and Family Considerations	Parental Health Expectations	0.418	0.064	0.029	[0.055, 0.074]	6
	Compatibility with Family Eating Practices	0.241	0.037	0.021	[0.031, 0.045]	14
	Parental Willingness to Provide	0.341	0.051	0.024	[0.044, 0.059]	9
Practical and Economic Value	Child-Appropriate Product Format	0.389	0.043	0.015	[0.038, 0.048]	11
	Convenience of Purchase and Use	0.340	0.037	0.012	[0.033, 0.041]	15
	Affordability and Value for Money	0.271	0.030	0.012	[0.026, 0.034]	17

Note: Mean weights were calculated from the individual priority vectors of the 35 retained participants using AIP. SDs and percentile-based 95% bootstrap CIs (10,000 resamples) are based on individual global weights. Rankings were determined using unrounded mean global weights.

Within the six dimensions, taste and flavor received the highest mean local weight for sensory appeal (0.476), followed by functional health benefits for nutritional and functional value (0.389) and food safety for safety and ingredient trust (0.457). Credibility of health claims ranked highest within packaging and product communication (0.428), parental health expectations within parental and family considerations (0.418), and child-appropriate product format within practical and economic value (0.389).

The variability estimates indicate that the global priority weights differed across individual participants. Food safety had a mean global weight of 0.113, with a standard deviation of 0.029 and a 95% bootstrap confidence interval from 0.104 to 0.123. The confidence intervals of several adjacent criteria overlapped, particularly taste and flavor, functional health benefits, and ingredient quality and naturalness. Their relative ordering should therefore be interpreted as a descriptive ranking within the present sample rather than evidence of clearly separated population-level priorities.

Local weights indicate the relative priority of criteria within their respective dimensions, whereas global weights incorporate both the local criterion weight and the weight of the corresponding higher-level dimension. Consequently, a criterion may rank first locally but occupy a lower position in the overall hierarchy. For example, credibility of health claims ranked first within packaging and product communication, but its mean global weight was 0.040 because the corresponding dimension received a comparatively low overall mean weight.

### 4.4. Comparison of Priority Weights Across Stakeholder Groups

Table 5 presents the mean priority weights, standard deviations, and rankings of the six dimensions across the five stakeholder groups. Safety and ingredient trust ranked first among parents, food and nutrition professionals, and child and family practitioners, and second among academic researchers and industry or product development practitioners. Nutritional and functional value ranked first among academic researchers, second among food and nutrition professionals and child and family practitioners, and third among parents and industry or product development practitioners.

**Table 5:** Mean Priority Weights, Standard Deviations, and Rankings of the Six Evaluation Dimensions across Stakeholder Groups.

Dimension	Parents	Academic Researchers	Food and Nutrition Professionals	Industry/Product Development	Child/Family Practitioners
Sensory Appeal	0.161 ± 0.052 (4)	0.169 ± 0.028 (3)	0.152 ± 0.021 (3)	0.255 ± 0.061 (1)	0.158 ± 0.027 (4)
Nutritional and Functional Value	0.197 ± 0.031 (3)	0.259 ± 0.032 (1)	0.267 ± 0.016 (2)	0.178 ± 0.016 (3)	0.201 ± 0.055 (2)
Safety and Ingredient Trust	0.248 ± 0.036 (1)	0.249 ± 0.038 (2)	0.280 ± 0.023 (1)	0.189 ± 0.025 (2)	0.269 ± 0.054 (1)
Packaging and Product Communication	0.068 ± 0.012 (6)	0.108 ± 0.021 (5)	0.079 ± 0.013 (6)	0.149 ± 0.018 (4)	0.077 ± 0.009 (6)
Parental and Family Considerations	0.213 ± 0.054 (2)	0.123 ± 0.034 (4)	0.120 ± 0.026 (4)	0.091 ± 0.019 (6)	0.194 ± 0.069 (3)
Practical and Economic Value	0.113 ± 0.040 (5)	0.092 ± 0.025 (6)	0.101 ± 0.019 (5)	0.139 ± 0.015 (5)	0.100 ± 0.028 (5)

Note. Values are mean ± SD based on the individual priority vectors within each stakeholder group. Rankings based on unrounded mean weights are shown in parentheses.

The leave-one-out sensitivity analysis showed that the complete rank ordering remained unchanged among parents, food and nutrition professionals, and industry or product development practitioners. Among academic researchers, nutritional and functional value and safety and ingredient trust exchanged the first and second positions in one of the six iterations. Among child and family practitioners, nutritional and functional value and parental and family considerations exchanged the second and third positions in three of the eight iterations, while safety and ingredient trust remained first throughout. Across the five groups, the maximum absolute change in any mean dimension weight ranged from 0.008 to 0.019. The observed rank changes were therefore confined to adjacent positions in two stakeholder profiles, with the remaining profiles retaining their complete ordering.

Several descriptive variations were apparent across the stakeholder profiles. Parental and family considerations ranked second among parents, whereas sensory appeal ranked first among industry or product development practitioners. Packaging and product communication ranked sixth in three profiles, fifth among academic researchers, and fourth among industry or product development practitioners. The standard deviations further indicated variation in individual priorities within each stakeholder group. These comparisons describe the participating stakeholders and do not establish differences between stakeholder populations.

Kendall’s coefficient of concordance was calculated from the rankings of the six dimensions across the five stakeholder profiles. The analysis yielded *W* = 0.739, χ^2^(5) = 18.486, and *p* = 0.002, indicating relatively high concordance in the overall ordering. Safety and ingredient trust and nutritional and functional value generally occupied higher positions, whereas packaging and product communication and practical and economic value tended to rank lower. Kendall’s *W* describes concordance among the five group-level ranking profiles; it does not measure agreement among individual participants or test differences between stakeholder populations.

## 5. Discussion

### 5.1. Core Priority Structure

The mean priority structure placed safety and ingredient trust, nutritional and functional value, and sensory appeal at the center of stakeholders’ evaluations of functional foods intended for children. Together, these dimensions accounted for 64.3% of the total priority weight. At the criterion level, food safety ranked first, followed by taste and flavor, functional health benefits, and ingredient quality and naturalness. This pattern indicates that participants distributed their priorities across protection from perceived risk, expected nutritional or health value, and anticipated sensory suitability. The prominence of safety and ingredient trust is consistent with functional food research showing that perceived health benefits, product credibility, and confidence in product characteristics contribute to consumer evaluations and acceptance [1,2,3,4,44]. The overlapping confidence intervals among several highly ranked criteria further suggest that these attributes formed a closely ranked set rather than a sharply differentiated sequence of priorities.

The prominence of safety and nutritional value can also be understood in relation to the context in which the products were evaluated. Because the questionnaire explicitly concerned foods intended for children, participants may have approached the comparisons with particular attention to product protection, ingredient reliability, and health relevance. From this perspective, the priority structure reflects the importance assigned to attributes associated with responsible food provision for children. It also shows that functional or nutritional benefits were evaluated in conjunction with confidence in the safety and integrity of the product.

Sensory appeal remained prominent despite the higher mean priorities assigned to safety and nutritional considerations. Taste and flavor ranked second among all 18 criteria, indicating that anticipated sensory qualities received substantial priority alongside safety and health-related attributes. Previous research has similarly identified taste, texture, and other sensory characteristics as important to children’s food responses and preferences [7,8,9,37]. The distribution of weights across safety, nutritional value, and sensory appeal indicates that adult stakeholder evaluations were not concentrated exclusively on either health-related attributes or anticipated sensory qualities. Product trustworthiness, nutritional relevance, and anticipated palatability instead emerged as the principal areas of emphasis within the specified evaluation framework.

### 5.2. Family and Product Contexts

Family-related considerations retained a meaningful position in the overall priority structure. Holsten et al. [16] and Bassett et al. [17] showed that children’s food choices are embedded in household food environments and negotiated through parental decision processes. In the present study, parental and family considerations ranked above practical and economic value and packaging and product communication. Parental health expectations received the highest local weight within the family dimension, followed by parental willingness to provide and compatibility with family eating practices. This pattern is consistent with research linking children’s eating behavior with family routines, parental food-choice motives, feeding practices, and household food environments [18,19,20].

Parental willingness to provide requires particular consideration because it may be informed by several other attributes included in the hierarchy, such as safety, nutritional value, sensory expectations, affordability, and convenience. Its weight should therefore not be interpreted as evidence that willingness operates independently of these attributes. Rather, it represents a provision-oriented judgment through which parents may integrate product characteristics with their health expectations and household circumstances. Retaining this criterion makes the family decision process visible within the hierarchy, while its conceptual proximity to other criteria should be acknowledged when interpreting the resulting weight.

The comparatively lower weights assigned to practical and communication-related dimensions do not indicate that these factors are irrelevant. Home food availability, parental practices, product usability, and compatibility with household routines can shape whether a product is incorporated into family diets [21,22,49]. The local priorities also provide a more differentiated interpretation of these dimensions. Credibility of health claims ranked first within packaging and product communication, suggesting that trustworthy information was prioritized over visual design when communication attributes were considered. Similarly, child-appropriate product format received the highest local weight within practical and economic value. Product form, convenience, affordability, information clarity, and claim credibility may therefore function as enabling conditions for routine household use, even though they were assigned lower global priorities than safety, nutrition, and sensory appeal [23,24].

### 5.3. Stakeholder Concordance and Descriptive Variation

The concordance analysis indicates that the five stakeholder groups shared broad priorities within the predefined evaluation framework. Safety and ingredient trust and nutritional and functional value occupied prominent positions across the mean group profiles, suggesting that their importance was not confined to a particular professional or household perspective. This pattern is consistent with functional food research linking consumer evaluations to perceived health benefits, product credibility, and confidence in product characteristics [1,2,3,4,44]. Research on foods intended for children also highlights parental consideration of nutritional contribution and the appropriateness of proposed health benefits [5,6]. The present findings identify safety and nutritional value as shared areas of emphasis among the participating stakeholders, although concordance in rankings does not imply identical weights or agreement among individual respondents.

Differences in emphasis were more apparent for sensory and family-related considerations. Sensory appeal ranked first among industry and product development practitioners, whereas parental and family considerations ranked second among parents. The industry profile is compatible with research emphasizing the relevance of consumer preferences, taste, texture, and product experience to food development [32,36,37,43]. The parent profile corresponds with research situating children’s food provision within household processes shaped by parental priorities, feeding practices, food availability, and established routines [17,18,19,20,21]. Studies of caregivers’ food decisions further illustrate the relevance of children’s anticipated responses and the circumstances of household provision [22,23,24]. These perspectives help contextualize the observed profiles, but the study did not test whether professional responsibilities or household roles explained the differences in priority weights.

The within-group standard deviations show that participants in the same stakeholder category did not assign identical priorities. AIP retained individual priority vectors before summarizing them as group means, consistent with the independent completion of the questionnaire [61]. The leave-one-out analysis showed that the complete ordering remained unchanged among parents, food and nutrition professionals, and industry or product development practitioners. Among academic researchers, safety and ingredient trust and nutritional and functional value exchanged the two highest positions in one iteration. Among child and family practitioners, nutritional and functional value and parental and family considerations exchanged the second and third positions, while safety and ingredient trust remained first. These changes qualify the interpretation of precise rankings: the broader areas of emphasis remained similar, but some adjacent positions were sensitive to individual judgments.

Taken together, the concordance and descriptive comparisons reveal shared priorities alongside differences in relative emphasis. Previous AHP applications in food research have used pairwise comparisons to examine the relative importance of sensory, consumer, technological, and practical attributes [33,34,35,36]. The present analysis applies this approach across professional and household perspectives, allowing the overall mean priorities to be considered alongside stakeholder-specific profiles and within-group variation. Its contribution lies in identifying where priorities converge and where their relative emphasis differs, without treating the profiles as distinct stakeholder typologies or established population differences. These patterns provide a basis for subsequent product-specific research examining whether similar priorities emerge when stakeholders evaluate concrete formulations and consumption contexts.

### 5.4. Implications for Product Development

The findings offer preliminary guidance for the early-stage development and evaluation of functional foods intended for children. The priority structure identifies food safety, ingredient quality and naturalness, nutritional relevance, and sensory appeal as attributes warranting attention during early-stage product evaluation. Decisions concerning ingredient selection and nutritional formulation may affect taste, texture, product format, and perceptions of product trustworthiness, making coordination across food safety, nutrition, sensory evaluation, and product development particularly important.

Ribeiro et al. [31] emphasized the value of incorporating children’s experiences into food co-creation, while Drake et al. [42] highlighted the importance of integrating sensory evaluation throughout product development. The present framework could be used as an initial screening tool to identify attributes that warrant attention before product-specific testing. It should not replace direct evaluation with children. Prototype development should include age-appropriate sensory testing and, where feasible, child-centered co-creation, together with parental assessments of safety, nutritional suitability, and household usability. This combined approach would allow developers to examine whether adult expectations correspond with children’s actual responses to a specific product.

Packaging and product communication may be most useful when they clarify and reinforce core product qualities. Evidence from other food contexts indicates that packaging and multisensory cues can influence product evaluation before consumption [43]. Within the present framework, credible health claims and clear product information appear more relevant than visual presentation alone, while child-appropriate formats and ease of use may facilitate household provision. Communication strategies should therefore present safety, ingredients, nutritional properties, and functional benefits clearly and credibly rather than rely primarily on visual appeal or broad health claims. This integrated interpretation is consistent with multicriteria food-development research in which technological, sensory, consumer, and practical requirements are considered jointly [35,36]. Given the exploratory and sample-specific design, these implications are best treated as priorities for further product-specific assessment rather than prescriptive industry standards.

### 5.5. Limitations and Future Research

The findings should be interpreted within the context of purposive recruitment of stakeholders with relevant professional or household experience in Taiwan. This approach incorporated informed perspectives, although the resulting profiles may not capture the full diversity of views within each stakeholder category. Standard deviations, bootstrap confidence intervals, and leave-one-out analyses helped characterize variation and sensitivity within the retained sample. These assessments inform the interpretation of the priority structure, but the group comparisons remain descriptive rather than population-level estimates. Extending the framework across professional settings, household circumstances, and cultural contexts would help assess the transferability of the observed priorities.

The evaluation concerned functional foods as a general category rather than specific products. Participants may therefore have considered different formulations, product formats, or consumption occasions when completing the comparisons. Moreover, the study assessed adult priorities without directly measuring children’s sensory responses or families’ purchasing and consumption behavior. The weights should consequently be understood as relative evaluative priorities rather than evidence of how particular attributes influence actual acceptance. Applying the framework to identifiable prototypes, together with age-appropriate sensory testing, child-centered co-creation, parent–child paired assessments, and observed food-choice measures, would allow adult expectations to be examined alongside children’s responses and household decisions.

The results are also conditional on the predefined hierarchy and operational definitions. Literature-based development and expert review supported the relevance and clarity of the framework, but some conceptual proximity remains among nutritional value, functional health benefits, age-appropriate nutritional suitability, and parental health expectations. Parental willingness to provide may likewise integrate judgments concerning attributes represented elsewhere in the hierarchy. The corresponding weights therefore do not establish independent contributions to food acceptance. Further refinement through formal content-validity assessment and respondent interviews could clarify how participants distinguish related criteria and whether alternative definitions or hierarchical arrangements produce comparable priority patterns.

Pairwise comparisons rely on subjective judgments expressed through a numerical scale. The fixed comparison order may also have introduced order or response-fatigue effects, neither of which was directly assessed. Although consistency screening addressed the internal coherence of the judgments, it did not establish their temporal stability. Future studies could randomize or counterbalance comparison order and use repeated assessments to examine whether priorities remain consistent over time. Sensitivity analyses involving alternative hierarchy specifications and aggregation procedures could further assess how methodological choices affect the resulting weights and rankings.

## 6. Conclusions

This study used a multi-stakeholder AHP framework to examine adult priorities for evaluating functional foods intended for children. Safety and ingredient trust, nutritional and functional value, and sensory appeal received the highest mean dimension weights. At the criterion level, food safety ranked first, followed by taste and flavor, functional health benefits, and ingredient quality and naturalness. Family, practical, and communication-related considerations received lower relative weights but remained part of the evaluation framework. These findings describe adult stakeholder priorities within the specified hierarchy rather than children’s directly measured food acceptance.

The stakeholder profiles showed broad concordance alongside variation in their relative emphasis. Sensory appeal ranked first among industry and product development practitioners, whereas parental and family considerations ranked second among parents. Safety and nutritional considerations occupied prominent positions across the profiles. These patterns identify shared priorities and differences in emphasis among the participating groups; they do not establish differences between stakeholder populations or explain the mechanisms underlying their judgments.

The framework offers a preliminary basis for identifying attributes that warrant attention during early-stage product evaluation. It brings safety, nutritional and functional value, sensory qualities, and family and product-use considerations into a common prioritization structure. Its practical value should be examined through product-specific development and testing, with adult evaluations considered alongside direct sensory assessment with children and evidence from family purchasing and consumption. This approach would help determine how the identified priorities correspond with responses to concrete products.

## Figures and Tables

**Table 1:** Dimensions, criteria, and operational definitions of the AHP evaluation framework.

Dimension	Criterion	Operational Definition
A. Sensory Appeal	A1. Taste and Flavor	The importance assigned to whether the overall taste and flavor profile of a functional food, including sweetness, saltiness, and other flavor characteristics, is likely to correspond with children’s preferences.
	A2. Texture and Mouthfeel	The importance assigned to whether texture and mouthfeel characteristics, including softness, crispness, smoothness, stickiness, and chewiness, are likely to be acceptable to children during eating.
	A3. Appearance and Color	The extent to which the color, shape, appearance, and overall visual presentation of a functional food are appealing to children.
B. Nutritional and Functional Value	B1. Nutritional Value	The importance assigned to the contribution of a functional food to children’s nutritional intake and dietary balance, including protein, vitamins, minerals, and appropriate levels of sugar, fat, and sodium.
	B2. Functional Health Benefits	The importance assigned to specific proposed health or physiological benefits beyond basic nutrition, such as support for digestion, immunity, cognition, or other functions relevant to children.
	B3. Age-Appropriate Nutritional Suitability	The importance assigned to whether the nutritional composition, nutrient density, serving design, and intended pattern of use are appropriate for children’s age and developmental stage.
C. Safety and Ingredient Trust	C1. Food Safety	The importance assigned to minimizing potential risks associated with ingredients, production, storage, preparation, and consumption when a functional food is intended for children.
	C2. Ingredient Quality and Naturalness	The importance assigned to the use of high-quality and natural ingredients and to limiting unnecessary artificial additives, colorants, preservatives, or highly processed components.
	C3. Certification and Quality Assurance	The importance assigned to credible safety or quality certifications, third-party verification, and quality assurance information that may strengthen confidence in the product.
D. Packaging and Product Communication	D1. Packaging and Visual Design	The importance assigned to packaging color, imagery, shape, and overall visual design in attracting attention and creating an appropriate product impression for children and parents.
	D2. Clarity of Product Information	The importance assigned to clear, complete, and understandable information concerning ingredients, nutrition, target age, instructions for use, and other product characteristics.
	D3. Credibility of Health Claims	The importance assigned to whether nutritional and health-related claims are credible, reasonable, and adequately supported by relevant information or scientific evidence.
E. Parental and Family Considerations	E1. Parental Health Expectations	The importance assigned to whether a functional food corresponds with parents’ expectations regarding their child’s nutrition, health, growth, and everyday dietary management.
	E2. Compatibility with Family Eating Practices	The importance assigned to whether a functional food can be incorporated into existing family eating habits, meal routines, shared meals, and everyday dietary practices.
	E3. Parental Willingness to Provide	The importance assigned to parents’ readiness to purchase, provide, or incorporate the functional food into their child’s diet as part of the family food-provision decision.
F. Practical and Economic Value	F1. Child-Appropriate Product Format	The importance assigned to whether the product form, size, serving amount, and mode of consumption correspond with children’s age, appetite, and practical eating needs.
	F2. Convenience of Purchase and Use	The importance assigned to whether a functional food is easy to purchase, access, store, carry, prepare, serve, and consume in everyday family life.
	F3. Affordability and Value for Money	The importance assigned to whether the product is affordable for households and offers reasonable value in relation to its nutritional benefits, functional benefits, safety, and overall quality.

**Table 2:** Composition and eligibility characteristics of stakeholder groups included in the AHP analysis.

Stakeholder Group	Eligibility and Relevant Background	n	%
Parents	At least one child under 12 years of age and direct involvement in household food purchase, selection, or provision	8	22.9
Academic researchers	At least 15 years of relevant academic or research experience	6	17.1
Food and nutrition professionals	At least 15 years of relevant professional experience in food science, nutrition, or related fields	6	17.1
Food industry/product development practitioners	At least 15 years of relevant industry, product development, culinary, or food service experience	7	20.0
Child health, education, family, or related practitioners	At least 15 years of relevant professional or practical experience in child health, education, family studies, health promotion, or related fields	8	22.9
Total		35	100.0

Note: The table reports the final analytical sample. A total of 39 questionnaires were completed, of which four were excluded because the pairwise comparison judgments did not satisfy the predefined consistency criterion of CR ≤ 0.10. Percentages were calculated using the final analytical sample of 35 participants.

## Data Availability

The original contributions presented in this study are included in the article. Further inquiries can be directed to the corresponding authors.
